# Characteristics, use of guideline–recommended medical therapies and clinical outcomes of patients with heart failure not enrolled in a quality registry: a comparison with the Swedish Heart Failure Registry

**DOI:** 10.1093/ehjqcco/qcaf019

**Published:** 2025-03-26

**Authors:** Ailema González-Ortiz, Paul Hjemdahl, Faizan Mazhar, Alessandro Bosi, Anne-Laure Faucon, Gianluigi Savarese, Lars H Lund, Juan Jesus Carrero

**Affiliations:** Department of Medical Epidemiology and Biostatistics, Karolinska Institutet, Stockholm, Sweden; Translational Research Center, Instituto Nacional de Pediatría, Mexico City, Mexico; Clinical Epidemiology Unit/Clinical Pharmacology, Department of Medicine Solna, Karolinska Institutet, Karolinska University Hospital, Stockholm, Sweden; Department of Medical Epidemiology and Biostatistics, Karolinska Institutet, Stockholm, Sweden; Department of Medical Epidemiology and Biostatistics, Karolinska Institutet, Stockholm, Sweden; Department of Medical Epidemiology and Biostatistics, Karolinska Institutet, Stockholm, Sweden; Department of Clinical Science and Education, Södersjukhuset; Karolinska Institutet, Stockholm, Sweden; Division of Cardiology, Department of Medicine, Karolinska Institutet, Stockholm, Sweden; Heart and Vascular and Neurology Theme, Karolinska University Hospital, Stockholm, Sweden; Department of Medical Epidemiology and Biostatistics, Karolinska Institutet, Stockholm, Sweden; Division of Nephrology, Department of Clinical Sciences, Danderyd Hospital, Danderyd, Sweden

**Keywords:** Heart failure, Quality registry, Guideline-recommended medical therapies, Clinical outcomes

## Abstract

**Introduction:**

Quality registries may involve specific inclusion criteria, detailed investigations, or selected hospitals and practitioners, which are not random. Whether the care and outcomes in quality registries are generalizable to the broader population is not well known. We here examine care indicators and outcomes in heart failure (HF) patients enrolled vs. non-enrolled in Swedish Heart Failure (SwedeHF) quality registry.

**Methods and results:**

Observational study of 90-day survivors after a HF in Stockholm (2012–2021). We linked health records from the Stockholm Creatinine Measurements project with SwedeHF. Participants enrolled in SwedeHF were compared to those non-enrolled, focusing on settings of care, use of guideline-recommended therapies, treatment adherence, dose titration, persistence, and outcomes. Analyses considered stratification by settings of management (primary care, cardiology-outpatient, and cardiology-inpatient care). We identified 48 374 incident HF cases of which 4878 (10%) were enrolled in SwedeHF within 90 days. Enrolled participants were younger, more often men and had fewer comorbidities than non-enrolled. Enrolled participants were more likely to initiate, persist and adhere to, and achieve higher dosages of guideline-recommended HF therapies (*P* < 0.05 for all). Enrolled participants were less likely to experience a major cardiovascular event [CV death, nonfatal myocardial infarction or stroke; HR 0.92, 95% confidence interval (CI) 0.86–0.99] and all-cause death (HR 0.87, 95% CI 0.82–0.92), but had similar rates of HF hospitalization (HR 1.03, 95% CI 0.94–1.15) compared to non-enrolled ones. Findings were similar across settings of management.

**Conclusion:**

Enrollment in the SwedeHF registry occurred in a minority of patients, and was associated with better adherence to guideline-recommended HF therapies and fewer major cardiovascular events and lower mortality. The generalizability of these HF registry findings to all HF patients was, however, limited.

## Graphical Abstract

**Graphical Abstract:**
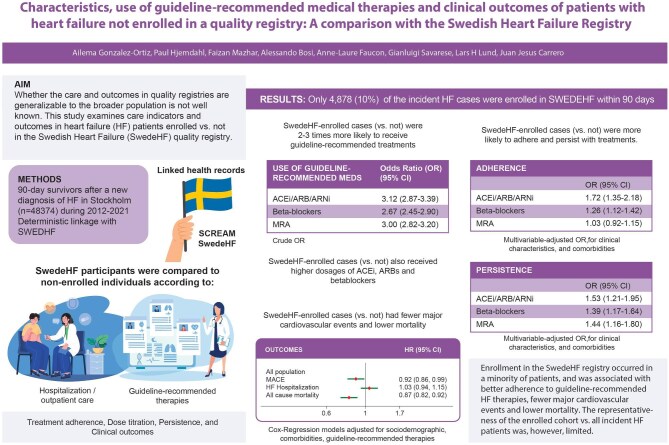

Key Learning Points **What is already known**:

- Quality registries may involve specific inclusion criteria, detailed investigations, or selected hospitals and practitioners, which are not random. Whether the care and outcomes in quality registries are generalizable to the broader population is not well known.

**What this study adds**:

- This study showed important differences in age, sex, and comorbidities between the modest proportion of patients with heart failure (HF) who were enrolled in the quality register SwedeHF and those who were not.
- Enrollment in SwedeHF was associated with increased initiation, persistence, and adherence to guideline-recommended drug treatments, and fewer major cardiovascular events.
- This study thus provides evidence of important care gaps in the large population of non-enrolled patients with HF that warrant correction. It also calls for caution in the generalization of findings from registries with non-complete coverage and illustrates the value of complete health systems data analyses.

## Introduction

Clinical quality registries systematically collect data on specific indicators of quality of care for various medical conditions, enabling the monitoring of care, providing feedback, benchmarking performance, identifying gaps, and reducing variation across centres. In cardiovascular medicine, several registries fulfill roles in quality improvement, reporting, benchmarking, standardization, and ensuring equal access to care.^1–3^ However, registries may impose specific inclusion/exclusion criteria or encompass hospitals and practitioners whose involvement is not random. Further, enrollment in a quality register likely exposes the caregiver to an intense checklist and comprehensive data collection protocol that ultimately may result in provision of better care. This introduces potential disparities in healthcare quality and outcomes between patients within and outside such registries and a need for studies of the generalizability of results from the quality registries.

Heart failure (HF) affects 2–3% of the adult population in developed countries, rising to ≥10% among people >70 years of age.^4,5^ The Swedish Heart Failure Registry (SwedeHF) is a voluntary clinical quality registry established in 2000 with the goal of improving the management of patients with HF by following guidelines regarding diagnosis and treatment more strictly. Although the coverage of SwedeHF has increased over the years, their 2021 annual report (last year of data coverage in the present study) describes capturing about 14% of incident HF cases in Sweden.^6^

In a previous study of HF cases from 2006 to 2013, we showed that those enrolled in SwedeHF had lower mortality rates compared to those who were not enrolled, and that better treatment with HF drugs partially explained their lower mortality.^7^ Limitations of that study included the lack of data from primary care, which is involved in the early identification and management of this population. In addition, updated HF guidelines from the European Society of Cardiology (ESC)^8,9^ and increased awareness of the benefits of evidence-based treatments could have reduced disparities in the clinical management of these patients.

The goal of this study is to identify healthcare gaps amenable to correction amongst under-studied populations. To that end, we analysed differences between SwedeHF-enrolled and non-enrolled patients on a more contemporary timeframe, focusing on key aspects of guideline-recommended medical therapy (use of recommended therapies, dose titration, adherence, and persistence) and their potential impact on health outcomes.

## Methods

### Data source

We used data from the Stockholm Creatinine Measurements (SCREAM) project, which contains healthcare utilization data from all residents of the region of Stockholm, Sweden.^10^ The Stockholm region provides universal and tax-funded healthcare to approximately a fourth of the population of Sweden. SCREAM contains complete information on demographics, healthcare utilization (including primary care), laboratory tests, dispensed drugs, diagnoses, and vital status. For the purpose of this study, SCREAM also contains linkage with SwedeHF. The Regional Ethical Review Board in Stockholm approved the study (reference 2017/793-31); informed consent was not deemed necessary since all data were de-identified by the Swedish Board of Health and Welfare.

### Study population

For this study, we included all adult (>18 years) residents of Stockholm who received, for their first time in their medical records, a diagnosis of HF between 2012 and 2021 in any source of care. We identified 55 395 new cases of HF through issued International Classification of Diseases (ICD) diagnostic codes in primary position (definitions in Supplementary material online, *Table S1*). After excluding 8 patients with missing information on age or sex, we cross-matched this cohort with registrations in SwedeHF (*n* = 11 231 registrations, 23% of total).

The ESC HF Guidelines^8,9,11^ recommend rapid institution and dose escalation of recommended therapies to achieve target doses. Because our goal was to compare care indicators, we focused on the immediate period post-diagnosis where therapies are to be started and up-titrated. Supplementary material online, *Figure S1* depicts the proportion of enrolments in SwedeHF by time since diagnosis, showing that over 60% of enrollments occurred within 90 days from the incident HF diagnosis. We chose this as a reasonable ascertainment period for enrollment as we were afraid that shorter periods would increase misclassification bias, and that longer periods would instead capture sicker patients who were referred to specialist care for worsening of their HF. We then required that patients in the identified population should survive the first 90 days in order to give the study groups an equal chance to initiate treatments. Specifically, we wanted to avoid that cases who died shortly after experiencing HF (and did not live enough to be enrolled in SwedeHF) biased the results. This resulted in the further exclusion of 7013 patients who died within the first 90 days post-diagnosis. At the end of the selection process, our study included 48 374 participants who were 90-day survivors of an incident HF. Of these, 4878 (10%) patients were enrolled in SwedeHF within 90 days (Supplementaty material online, *Figure S2*).

### Study exposure

The study exposure was enrollment in SwedeHF (vs. non enrollment). Because care processes may differ by HF severity and care provider, our main stratifier considered the setting in which the HF was identified and managed, as follows: (i) *cardiology-managed inpatient/hospitalized cases* defined as cases that during the 90 days of ascertainment had at least one recording of hospitalization for HF in a cardiology clinic; (ii) *cardiology-managed outpatient cases*: defined as cases that during the 90 days of ascertainment had at least one diagnosis of HF in cardiology-specialist care; and (iii) *primary care-managed cases* defined as cases that during the 90 days of ascertainment solely had HF diagnoses issued in primary care. Baseline characteristics were estimated at the date of HF diagnosis, and the follow-up started after the 90-day period. In this study there are a series of concatenated analyses, each of them with a different index date to avoid immortal time biases.

### Study covariates

Study covariates were calculated at the time of HF diagnosis and considered demographics (age and sex), socioeconomic characteristics (civil status and highest attained education), comorbidities, laboratory values, and medications. Comorbidities were defined by the presence of relevant ICD codes prior to the index date (see listing and definitions in Supplementary material online, *Table S1*). We estimated the patient's ejection fraction with a SwedeHF-based algorithm^12^ to estimate the likelihood of having preserved or reduced ejection fraction based on the patient’s comorbidity profile (see description and modifications to fit our data in Supplementary material online, Page 4). Outpatient plasma creatinine tests were used to estimate glomerular filtration rate (eGFR),^13^ and chronic kidney disease (CKD) was defined as having eGFR <60 mL/min/1.73 m^2^.^14^ Ongoing use of key cardiovascular-related medications was ascertained by filled prescriptions at the time of or within 6 months prior to the HF diagnosis (see listing and definitions in Supplementary material online, *Table S2*).

### Use of guideline-recommended therapies and medication-related outcomes

We evaluated the use of ESC guideline-recommended medical therapies available during the periods evaluated in this analysis.^8,9,11^ Guidelines from 2012 recommended angiotensin-converting enzyme inhibitors(ACEis) or angiotensin receptor blockers (ARBs), beta-blockers, and mineralocorticoid receptor antagonists (MRAs). In 2016, angiotensin receptor neprilysin inhibitors (ARNIs) were also included. We did not evaluate the use of Sodium-glucose cotransporter-2 inhibitors as the indication for HF had not yet been approved in Sweden during the data collection period.

The first medication-related outcome was *treatment initiation*, defined by records of pharmacy dispensations in the national prescribed drug register^15^ which has complete coverage of all dispensed prescriptions at Swedish pharmacies. The index date of these analyses was the date of HF diagnosis, and we explored initiation of treatments within 30, 90, and 180 days from the index date. Some patients were already using the medications of interest prior to the HF diagnosis (i.e. prevalent medication users). For these patients, we estimated the expected date for a new pharmacy fill based on the pill supply from the last dispensation prior to SwedeHF registration, and treatment initiation was defined as a filled prescription after the end of the estimated pill supply.

The following medication-related outcomes were dependent on treatment start and enrollment, and their index dates thus vary: *treatment adherence*  and  *persistence* were calculated for the first year and first 3-years of therapy (or until censoring), and the index date was the date of first dispensation of the medication of interest after the incident HF diagnosis. We determined adherence by assessing the proportion of days covered (PDC), a method that considers the number of consecutive fills and the distance (in days) between them. The expected duration of the fill (i.e. the number of days that one package of medication would last) was estimated through the average frequency of dispensing for each single formulation in the study population. Low adherence was defined as a PDC <80%.^16,17^ Non-persistence (i.e. discontinuation of therapy) was defined by the absence of a new dispensation during at least 120 days after the end of the last estimated pill supply.

Our final medication-related outcome was *medication dose*. Using the defined daily doses (DDDs,^18^ in mg of active principle) for each single dispensed medication, the number of pills of the package and the time it takes (in days) to fill the next prescription, we estimated the doses in mg consumed per day. Because our study hypothesis is that SwedeHF enrolled participants are likely to receive more guideline-compliant treatment patterns (i.e. higher treatment dosages) than non-enrolled, this outcome was calculated with the two immediate pharmacy fills after 90 days from HF diagnosis. Thus, all participants had the same follow-up since HF diagnoses and all enrolled participants by day 90 were equally considered.

### Health outcomes

We also explored if enrollment in SwedeHF (vs. no enrollment) was associated with differences in health outcomes. The outcomes considered were all-cause death, major adverse cardiovascular events (MACEs, a composite of cardiovascular death, hospitalization for myocardial infarction or ischaemic stroke) and HF (re-)admission. Because our patient selection was conditional on surviving the first 90-days post diagnosis, the index date of this analysis was at day 90 from the diagnosis of HF. Patients were followed until an event, emigration from the region, end of follow up (31st December 2021), or death, whichever occurred first (outcome definitions listed in Supplementary material online, *Table S3*).

### Statistical analysis

Values are expressed as mean and standard deviation (SD) for continuous variables with normal distribution, median, and interquartile range (IQR) for non-normally distributed variables, and percentage for categorical variables.

We first compared the clinical characteristics and setting of care between SwedeHF-enrolled and non-enrolled participants using univariable logistic regression with enrolment in SwedeHF as the dependent variable. In this analysis, the index date was the date of HF diagnosis.

Next, differences in medication-related outcomes between SwedeHF-enrolled and non-enrolled participants were explored through logistic regression, adjusting when applicable for differences in patient characteristics. Absolute risks were computed for adherence and persistence, taking into account the competing risk of death or migration using the Aalen–Johansen estimator.

Finally, the association with health outcomes was assessed through multivariable-adjusted Cox regression models. Multiplicative interaction tests explored possible outcome differences according to setting of management.

In order to evaluate if our ascertainment window of 90 days to define enrolment in SwedeHF introduced misclassification bias, we performed a sensitivity analysis where we repeated key main analyses considering a longer ascertainment window of 180 days from the incident HF diagnosis (*n* = 45 225). In addition, we performed analyses stratified by estimated ejection fraction strata. All statistical analyses were conducted using R version 3.5.1 and STATA software (version 17.1; Stata Corp, College Station, TX, USA).

## Results

### Characteristics at HF diagnosis

Out of 48 374 incident HF cases occurring in Stockholm during 2012–2021, 4 878 (10%) were enrolled in SwedeHF within 90 days. The characteristics of non-enrolled and enrolled participants are shown in *Table 1*. Enrolled participants were younger and more often male. Enrolled participants were also less likely to have preserved ejection fraction and comorbid conditions like hypertension, CKD, diabetes, chronic lung disease, anaemia, and other types of cardiovascular diseases (CVDs). However, enrolled participants were more likely to have suffered a myocardial infarction and to have undergone a percutaneous coronary intervention. Ongoing treatments at the time of HF diagnosis also varied: enrolled participants were more likely to be on MRAs at the time of incident HF diagnosis, but less likely to receive other CVD-treatments like diuretics, digoxin, anticoagulants or antiplatelets than non-enrolled participants. Finally, enrolled participants were more likely to be identified and managed in cardiology, with a very small representation of patients managed in primary care settings.

**Table 1:** Sociodemographic and clinical characteristics or adults with incident HF in Stockholm, Sweden, during 2012–2021, overall and by enrollment in the SwedeHF registry, along with univariable Odds Ratio of enrollment (vs. non-enrollment)

	All HF cases	Non-enrolled	Enrolled in SwedeHF	Odds ratio of enrollment
	*n* = 48 374	*n* = 43 496	*n* = 4878	(95% CI)
Sociodemographic characteristics %				
Women	48	49	36	0.58 (0.54–0.61)
Age years mean (SD)	76(13)	77(13)	70(13)	0.96 (0.96–0.96)
>75 years old	62	64	40	0.37 (0.35–0.39)
*Living status*				
Living alone	56	57	48	0.70 (0.66–0.75)
Living with someone	44	43	51	1.42 (1.34–1.51)
Unknown	0.4	0.4	0.6	–
*Highest attained education*				
Compulsory school	30	31	26	0.77 (0.72–0.82)
Secondary school	40	40	43	1.12 (1.05–1.19)
University	26	26	29	1.19 (1.11–1.27)
*Estimated ejection fraction category*				
HFpEF (EF ≥ 50%)	49	51	35	0.52 (0.49–0.56)
HFmrEF or HFrEF (EF < 50%)	39	38	46	1.39 (1.31–1.47)
Unknown	12	11	19	–
Setting of identification and care %				
Cardiology inpatient care	48	47	60	1.67 (1.57–1.77)
Cardiology outpatient care	24	23	38	2.07 (1.94–2.20)
Primary care	28	31	3	0.07 (0.06–0.08)
Comorbid conditions and history of procedures %				
Hypertension	72	74	58	0.49 (0.46–0.52)
Chronic kidney disease	32	33	23	0.59 (0.56–0.64)
Diabetes mellitus	24	25	22	0.87 (0.81–0.93)
Myocardial infarction	16	16	17	1.12 (1.03–1.21)
Percutaneous coronary intervention	12	12	14	1.27 (1.17–1.37)
Coronary artery bypass graft	4	4	4.5	1.12 (0.97–1.30)
Angina pectoris (within 3 years)	21	21	17	0.77 (0.72–0.84)
Stroke or TIA including haemorrhagic	19	20	13	0.60 (0.56–0.66)
Any severe bleed except intracranial	3	3	2	0.60 (0.49–0.72)
Peripheral artery disease	12	12	10	0.83 (0.76–0.91)
Aortic stenosis	6	6	5	0.75 (0.66–0.85)
Aortic valve surgery	2	2	2	0.95 (0.78–1.17)
Atrial fibrillation	41	41	35	0.78 (0.73–0.82)
Lung disease	32	32	28	0.81 (0.76–0.87)
COPD	17	18	13	0.69 (0.63–0.75)
Recent anaemia (1 year)	16	17	13	0.73 (0.67–0.79)
*HF specific medications* %				
*ACEi/ARB/ARNi*	58	58	58	1.02 (0.97–1.09)
*Beta-blocker*	61	61	58	0.88 (0.83–0.93)
*MRA*	9	9	11	1.28 (1.16–1.40)
*Other cardiovascular medications* %				
*Diuretics*	47	48	38	0.67 (0.63–0.71)
*Digoxin*	6	6	5	0.83 (0.72–0.95)
*Anticoagulants*	33	34	31	0.91 (0.85–0.97)
*Statins*	37	37	36	0.98 (0.92–1.04)
*Aspirin/antiplatelets*	35	35	32	0.87 (0.82–0.93)
*Calcium channel blockers*	29	30	23	0.69 (0.64–0.74)

Categorical variables are presented as percentages. Continuous variables are presented as mean (standard deviation) SD, standard deviation; TIA, transient ischaemic attack; and COPD, chronic obstructive pulmonary disease.

HF was categorized as HF with preserved ejection fraction (HFpEF: EF ≥ 50%) or reduced ejection fraction (HFrEF or HFmrEF: EF < 50%), following a SwedeHF-based algorithm to estimate ejection fraction (EF) from claims data.^12^

Characteristics of the included participants stratified by the setting of care is shown in Supplementary material online, *Table S4*. The above patterns varied by care setting, with outpatient and inpatient cardiology showing higher enrollment rates and greater adherence to treatments, while primary care had lower enrollment and fewer documented differences. This highlights potential selection biases favouring healthier or better-managed patients for enrollment.

### Use of guideline-recommended therapies

Within all horizons explored (30, 90, and 180 days from diagnosis), SwedeHF-enrolled participants were 2–3 times more likely to initiate or continue guideline-recommended treatment with ACEi/ARB/ARNi, beta-blockers or MRA (*Table 2*). For example, in absolute terms, 86% and 41% of SwedeHF-enrolled patients received ACEi/ARB/ARNi and MRA within 90 days from diagnosis, respectively, whereas the corresponding proportions in non-enrolled patients were 60 and 19%. Analyses by setting of care showed that SwedeHF-enrolled patients consistently had higher rates of medication initiation. The odds ratios show that enrollment significantly increased the likelihood of receiving ACEi/ARB/ARNi, beta-blockers, and MRA in all settings, and that the primary care setting had the largest relative differences in initiation rates (Supplementary material online, *Table S5*).

**Table 2:** Unadjusted Odds Ratio of use of guideline-recommended within 30, 90, and 180 days from diagnosis and by enrollment in SwedeHF

	Within 30 days from diagnosis			Within 90 days from diagnosis			Within 180 days from diagnosis		
Medications	Non-enrolled, %	SwedeHF-enrolled,%	Odds ratio(95% CI)	Non-enrolled, %	SwedeHF-enrolled, %	Odds ratio(95% CI)	Non-enrolled, %	SwedeHF-enrolled, %	Odds Ratio(95% CI)
**ACEi/ARB/ARNi**	48	68	2.30 (2.16-2.45)	66	86	3.12 (2.87–3.39)	72	90	3.63 (3.29–4.00)
**Beta-blockers**	50	66	1.93 (1.82-2.06)	70	86	2.67 (2.45–2.90)	76	91	3.10 (2.81–3.42)
**MRA **	13	28	2.56 (2.39-2.74)	19	41	3.00 (2.82–3.20)	22	47	3.01 (2.83–3.20)

ACEi, angiotensin-converting enzyme inhibitor; ARB, angiotensin receptor blocker; ARNi, angiotensin receptor—neprilysin inhibitor; MRA, mineralocorticoid receptor antagonist. *ARNi initiations were considered for the period 2016–2021.

For most single medications studied, the median dispensed dose was higher in SwedeHF-enrolled participants compared to non-enrolled (*Table 3*). The doses were, however, lower than the recommended target doses in both groups. We observed the same trend across all settings of care.

**Table 3:** Median daily doses (mg/d) for dispensed guideline-recommended single medications in SwedeHF-enrolled and non-enrolled participants

	Population *N*	DDD	Recommendedtarget dose*	Non-enrolled patients*N*, median mg/day (IQR)	SwedeHF-enrolled patients* N*, median mg/day (IQR)	*P*-value
*Mineralocorticoid receptor antagonists*						
Spironolactone	4746	75 mg	50 mg o.d.	*n* = 376726.9 (21.9–37.9)	*n* = 97926.9 (22.3–38.5)	0.74
Eplerenone	1525	50 mg	50 mg o.d.	*n* = 101130.9 (24.3*–*47.2)	*n* = 51438.9 (26.0–52.6)	<0.001
*Beta-blockers*						
Metoprolol	14 616	150 mg	200 mg o.d.	*n* = 12 80595.2 (51.0–166.7)	*n* = 1811119.0 (67.6–197.3)	<0.001
Bisoprolol	9461	10 mg	10 mg o.d.	*n* = 78365.5 (2.9–9.9)	*n* = 16256.9 (3.8–10.5)	<0.001
Carvedilol	397	37.5 mg	25 mg b.i.d.	*n* = 33726.1 (14.6–46.9)	*n* = 6024.7 (13.7–46.8)	0.94
*ACEi/ARB/ARNi* **						
Enalapril	6522	10 mg	10-20 mg b.i.d.	*n* = 569711.6 (6–20)	*n* = 82517.2 (9.4–23.3)	<0.001
Ramipril	6150	2.5 mg	10 mg o.d.	*n* = 50436.8 (3.7-10.2)	*n* = 11078.9 (5.4-11.1)	<0.001
Candesartan	6905	8 mg	32 mg o.d.	*n* = 584016.3 (8.3*–*30.7)	*n* = 106524.5 (13.2–35.6)	<0.001
Losartan	3248	50 mg	150 mg o.d.	*n* = 298466.2 (44.1–105.4)	*n* = 26484.5 (45.7–120.1)	0.005
Valsartan/Sacubitril	398	2 doses	97/103 mg b.i.d.	*n* = 1752.3 (1.9–3.4)	*n* = 2222.4 (1.9–3.7)	0.39

mg, miligrams; o.d. (once daily); b.i.d. (twice daily).

* Target doses are dose recommended for the treatment of patients with HFrEF according to the 2016 ESC Guidelines^11^;

** Other ACEi or ARBs and combinations with diuretics were prescribed to fewer than 1% of the patients and not considered in this analysis.

The persistence and adherence to all guideline-recommended medical therapies was higher among SwedeHF-enrolled participants compared to non-enrolled ones except for MRA (*Table 4* and Supplementary material online, *Table S6*). Interaction tests suggested heterogeneity for the persistence of MRA, being better in patients managed and identified in cardiology outpatient care compared to other settings (Supplementary material online, *Table S6*).

**Table 4:** Multivariable-adjusted odds ratios and 95% (CI) for the adherence (Panel A) and persistence (Panel B) to therapies in SwedeHF-enrolled vs. non-enrolled participants, overall and by setting of care

			By setting of care			
		Overall*n* = 48 374		Outpatient cardiology*n* = 11 623	Inpatient cardiology*n* = 23 286	Primary care*n* = 13 465
*Panel A. One year adherence to guideline-recommended medical therapies*						
	**% adherent enrolled/non-enrolled**	**OR (95% CI)**	**OR (95% CI)**	**OR (95% CI)**	**OR (95% CI)**	***P* for interaction**
*ACEi/ARB/ARNi*	94/88	1.72 (1.35–2.18)	1.72 (1.35–2.19)	1.81 (1.50–2.17)	2.98 (1.19–7.49)	0.25
*Beta-blocker*	67/50	1.26 (1.12–1.42)	1.25 (1.11–1.41)	1.26 (1.15–1.38)	1.38 (0.90–2.10)	0.21
*MRA*	39/39	1.03 (0.92 –1.15)	1.42 (1.15–1.74)	0.93 (0.82–1.06)	1.06 (0.47–2.37)	0.01
*Panel B. One year persistence to guideline-recommended medical therapies*						
	**% persistent enrolled/non-enrolled**	**OR (95% CI)**	**OR (95% CI)**	**OR (95% CI)**	**OR (95% CI)**	***P* for interaction**
*ACEi/ARB/ARNi*	94/87	1.53 (1.21–1.95)	1.55 (1.22–1.98)	2.08 (1.7–2.46)	1.50 (0.71–3.16)	0.32
*Beta-blocker*	86/79	1.39 (1.17–1.64)	1.38 (1.17–1.62)	1.65 (1.46–1.87)	2.53 (1.30–4.94)	0.58
*MRA*	69/58	1.44 (1.16–1.80)	1.36 (1.22–1.51)	1.33 (1.17–1.53)	1.37 (0.61–3.10)	0.32

ACEi, angiotensin-converting enzyme inhibitor; ARB, angiotensin receptor blocker; ARNi, angiotensin receptor—neprilysin inhibitor; and MRA, mineralocorticoid receptor antagonist. *We create a model per each medication, adjusted for clinical characteristics (age, gender, and when applicable setting of care), and comorbidities (hypertension, chronic kidney disease, diabetes mellitus, myocardial infarction, percutaneous coronary intervention, coronary artery bypass graft, angina pectoris, stroke, severe bleed, aortic stenosis, aortic valve surgery, atrial fibrillation, lung disease, COPD, anaemia).

#### Health outcomes

**Figure 1:**
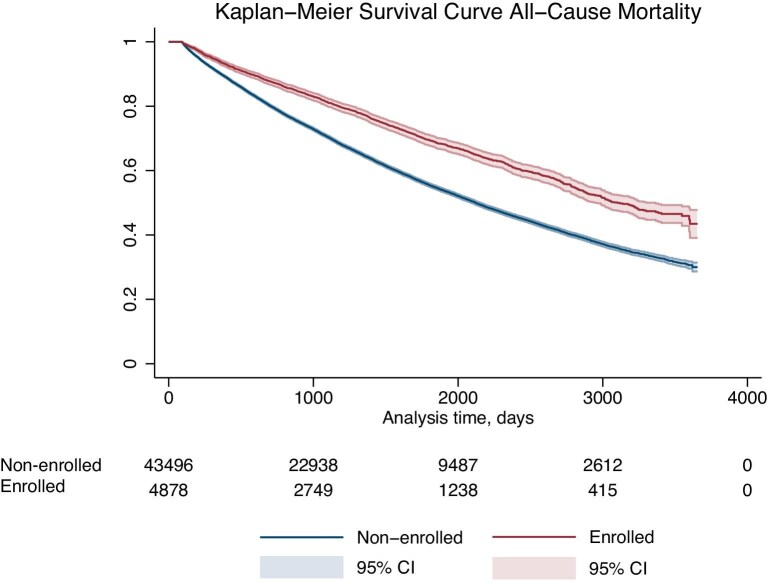
Kaplan–Meier Survival curve for all-cause mortality by enrollment in the SwedeHF registry; non-enrolled individuals (lower curve) and enrolled individuals (upper curve). The shaded areas around the survival curves represent 95% confidence intervals (CI) for each group.

During a median follow-up of 2.9 (IQR 1.4–5.1) years, we observed 13.045 MACE events (27%), 16 951 HF readmissions (35%), and 19 065 deaths (39%). In fully-adjusted models and compared to non-enrolled participants, those enrolled in SwedeHF had a 8% lower rate of MACE [HR 0.92, 95% confidence interval (CI) 0.86–0.99 *P* = 0.02] due to fewer CV deaths (HR 0.81, CI 0.66–0.98 *P* = 0.03) and myocardial infarctions (HR 0.86, 95% CI 0.77–0.98 *P* = 0.02) and a tendency towards fewer ischaemic strokes (HR 0.99, 95% CI 0.88–1.11 *P* = 0.89), as well as a lower rate of all-cause death (HR 0.87; 95% CI 0.82–0.92 *P* < 0.01). However, the rates of HF (re-)admission did not differ between enrolled and non-enrolled patients (*Table 5*). Multiplicative interaction tests suggested a potential for heterogeneity in the association between SwedeHF enrollment and health outcomes across settings: there were lower rates of MACE for SwedeHF-enrolled participants, with a magnitude of rate reduction slightly larger for those managed in cardiology outpatient care; other study outcomes followed similar tendencies, but they were affected by broader confidence intervals. *Figure 1* shows a higher probability of survival over time for enrolled individuals.

**Table 5:** Adverse health outcomes associated with enrollment in SwedeHF (vs. non-enrollment)

				By Setting of care			
	Overall*n* = 48 374			Outpatient cardiology *n* = 11 623	Inpatient cardiology *n* = 23 286	Primary care*n* = 13 465	
	*No. events/cases*Non-enrolled	*No. events/cases*Enrolled	HR (95% CI)	HR (95% CI)	HR (95% CI)	HR (95% CI)	*P* for interaction
***MACE***	11 970/43 496	1075/4878	0.92(0.86–0.99)	0.89(0.78–1.01)	0.94(0.87–1.01)	0.97(0.70–1.35)	<0.01
**HF hospitalization**	15 065/43 496	1886/4878	1.03(0.94–1.15)	1.06 (0.97–1.19)	1.22(1.15–1.29)	1.29(0.97–1.73)	<0.01
**All-cause mortality**	17 713/43496	1352/4878	0.87(0.82–0.92)	0.96(0.86–1.08)	0.85(0.79–0.92)	0.85(0.64–1.12)	<0.01

Output from Cox-Regression models adjusted for sociodemographic variables (age, sex), history of comorbidities (hypertension, chronic kidney disease, diabetes mellitus, myocardial infarction, percutaneous coronary intervention, coronary artery bypass graft, angina pectoris, stroke, severe bleed, aortic stenosis, aortic valve surgery, atrial fibrillation, lung disease, COPD, anaemia), guideline-recommended therapies (ACEi/ARB/ARNi, beta-blocker, MRA) and, when applicable, setting of care.

### Sensitivity and subgroup analyses

Increasing our ascertainment window for SwedeHF enrollment to 180-days post-diagnosis identified 45 225 survivors, of which 5 249 (11%) where enrolled in SwedeHF. We confirmed similar trends for guideline-recommended therapy initiation, adherence, and persistence (Supplementary material online, *Tables S7–S9*). Additionally, we observed similar associations with health outcomes from day 180 onwards (Supplementary material online, *Table S10*).

Subgroup analyses among individuals with ejection fraction below 50% showed similar differences in admission characteristics between enrolled/non-enrolled participants, and similar trends regarding the initiation, adherence, and persistence to guideline-recommended (Supplementary material online, *Tables S11–S13*). Although smaller sample sizes yielded at times broader and non-statistically significant confidence intervals, the magnitudes of the odds ratios were similarly elevated as in our main analysis.

## Discussion

The present study, conducted in a complete regional healthcare system, shows important differences between the modest proportion of patients with HF who were enrolled in the quality register SwedeHF and those who were not. Enrolled patients were younger, more often male, and had fewer cardiovascular and other comorbidities than non-enrolled patients. Enrollment in SwedeHF was associated with increased initiation, persistence and adherence to guideline-recommended HF drug treatment, and fewer major cardiovascular events. These results expand previous research demonstrating the effectiveness of structured care models in enhancing patient outcomes through better treatment and closer monitoring and follow-up, potentially explaining better health outcomes.^19–21^

We focused on differences between patients who were enrolled in SwedeHF within 90 days of an incident HF diagnosis in order to see which advantages a structured enrolment in a quality registry might afford for newly diagnosed patients. Patients who were enrolled in SwedeHF at later time points were probably patients with HF who were referred to specialist care for optimization of treatment, including device therapy due to worsening of their HF, and these patients probably had a more complicated history and more confounding factors than newly diagnosed patients.

While early enrollment in SwedeHF was linked to a 15% reduction in MACE, including a lower cardiovascular mortality, and a 12% reduction of all-cause mortality we observed no overall differences in HF hospitalization between enrolled and non-enrolled patients. This suggests that while registries support adherence to guidelines and treatment adherence, other factors, such as baseline comorbidities and treatment settings, may also heavily influence soft outcomes such as hospitalization. We note that the coverage of enrollment in SWEDEHF was much larger for cardiology units than for primary care units.^22–25^

Early enrolment in SwedeHF was found for only 10% of the HF population and these patients differed from non-enrolled patients in many respects apart from being, on average, 7 years younger and more often male. Myocardial infarction and coronary revascularization were more common among enrolled patients but hypertension, diabetes, and a number of CVDs were less common among enrolled patients. Thus, patients enrolled in SwedeHF are poorly representative of the entire HF population.

Guidelines recommend rapid institution and dose escalation of HF drug treatment to achieve target doses for the different drugs.^8,9,11^ We found that the dosages of ACEi, ARBs and beta-blockers were higher among SwedeHF-enrolled patients, but the guideline-recommended target doses for these drugs and for MRAs were still not reached. Higher ages and more comorbidities among non-enrolled patients may have contributed to the differences seen and the non-enrolled group probably had a greater percentage of patients with HFpEF with fewer indications, or other indications for their CV drug treatment. However, participation in the quality registry most likely increased the emphasis on dose titration among those with heart failure with reduced ejection fraction (HFrEF). Increased adherence among enrolled patients suggests that clinics/clinicians participating in the registry follow their patients more closely.

Quality registries, such as SwedeHF, could play a crucial role in monitoring and enhancing cardiovascular care.^20^ However, the coverage of this quality register needs to be improved so that results from it can be generalized. Future efforts should consider expanding registry access or introducing registry-like follow-up in broader clinical settings, including primary care, to address disparities. Integrating such care structures with real-time feedback systems might standardize HF management across all healthcare settings, potentially improving outcomes for patients traditionally excluded from quality registries. Quality registries should strive to include a population which is as complete and/or representative as possible. When completeness is not feasible, they should systematically assess the potential impact of their inclusion and exclusion criteria on the validity and generalizability of their findings. This is important to evaluate how well the results remain applicable to broader populations. Evidence from systematic reviews suggests that clinical quality registries may play a significant role in improving health outcomes and reducing healthcare costs, provided that they are representative.^1^

Our study has several strengths, including the complete capture of all HF cases in the region and the capacity to identify and separate management in primary vs. cardiology care. We also have a robust study design and methodology to ascertain indicators of pharmacotherapy. A limitation of our study is the lack of information on blood pressure, body mass index, or smoking habits which may influence HF severity and treatment efficacy. We also acknowledge the lack of ejection fraction and ICD-10 codes for HF ejection fraction subtypes which were introduced only recently. There was probably a greater percentage of patients with EF <50% and thus an indication for the guideline-recommended therapies in the SwedeHF enrolled group which comprised a greater proportion of male patients and younger patients than the non-enrolled group. We attempted to circumvent this by using a SwedeHF-based algorithm to estimate EF.^12^ Finally, our findings represent care practices in the region of Stockholm, and extrapolation to other regions in Sweden or other countries should be done with caution.

To conclude, this study shows that enrolment in a HF quality registry is not at random, and that this is associated with better provision of guideline-recommended therapies, including higher initiation rates, higher dosages, and greater adherence and persistence, as well as a lower risk of MACE but not of HF hospitalization. It is unclear if differences are explained by more stringent clinical praxis of centres reporting to the quality registry, or by the structured care model imposed by undertaking the registration of data. From a clinical perspective, our study evidences important care gaps in the large population of non-enrolled patients with HF that warrant correction. It also calls for caution in the generalization of findings from registries with non-complete coverage, and illustrates the value of complete health systems data analyses.

## Supplementary Material

qcaf019_Supplemental_File

## Data availability

The data may be shared on reasonable request to Prof. Carrero (juan.jesus.carrero@ki.se) for academic research collaborations that comply with the General Data Protection Regulation as well as national and institutional ethics regulations and standards.

## References

[1] Hoque DMdE, Kumari V, Hoque M, Ruseckaite R, Romero L, Evans SM. Impact of clinical registries on quality of patient care and clinical outcomes: a systematic review. PLoS One 2017;12:e0183667. 10.1371/journal.pone.0183667 28886607 PMC5591016

[2] Anell A . The public–private pendulum—patient choice and equity in Sweden. N Engl J Med 2015;372:1–4. 10.1056/NEJMp1411430 25551523

[3] Pol T, Karlström P, Lund LH. Heart failure registries—future directions. J Cardiol 2024;83:84–90. 10.1016/j.jjcc.2023.10.006 37844799

[4] Mosterd A, Hoes AW. Clinical epidemiology of heart failure. Heart 2007;93:1137–1146. 10.1136/hrt.2003.025270 17699180 PMC1955040

[5] Savarese G, Becher PM, Lund LH, Seferovic P, Rosano GMC, Coats AJS. Global burden of heart failure: a comprehensive and updated review of epidemiology. Cardiovasc Res 2023;118:3272–3287. 10.1093/cvr/cvac013 35150240

[6] https://www.ucr.uu.se/rikssvikt/om-rikssvikt/arsrapporter (Nov) 2024.

[7] Lund LH, Carrero J‐.J, Farahmand B, Henriksson KM, Jonsson Å, Jernberg T et al. Association between enrolment in a heart failure quality registry and subsequent mortality-a nationwide cohort study. Eur J Heart Fail 2017;19:1107–1116. 10.1002/ejhf.762 28229520

[8] Ponikowski P, Voors A A, Anker D S, Bueno H, Cleland GF J, Coats JS A et al. 2016 ESC Guidelines for the diagnosis and treatment of acute and chronic heart failure: the Task Force for the diagnosis and treatment of acute and chronic heart failure of the European Society of Cardiology (ESC). Developed with the special contribution of the Heart Failure Association (HFA) of the ESC. Eur J Heart Fail 2016;18:891–975. 10.1002/ejhf.592 27207191

[9] McMurray JJ, Adamopoulos S, Anker D S, Auricchio A, Böhm M, Dickstein K et al. ESC Guidelines for the diagnosis and treatment of acute and chronic heart failure 2012: the Task Force for the Diagnosis and Treatment of Acute and Chronic Heart Failure 2012 of the European Society of Cardiology. Developed in collaboration with the Heart Failure Association (HFA) of the ESC. Eur Heart J 2012;33:1787–1847. 10.1093/eurheartj/ehs104 22611136

[10] Carrero JJ, Elinder CG. The Stockholm CREAtinine Measurements (SCREAM) project: fostering improvements in chronic kidney disease care. J Intern Med 2022;291:254–268. 10.1111/joim.13418 35028991

[11] Ponikowski P, Voors AA, Anker SD, Bueno H, Cleland JGF, Coats AJS et al. 2016 ESC guidelines for the diagnosis and treatment of acute and chronic heart failure. Rev Esp Cardiol (English Edition) 2016;69:1167. 10.1016/j.rec.2016.11.005 27894487

[12] Uijl A, Lund LH, Vaartjes I, Brugts JJ, Linssen GC, Asselbergs FW et al. A registry-based algorithm to predict ejection fraction in patients with heart failure. ESC Heart Fail 2020;7:2388–2397. 10.1002/ehf2.12779 32548911 PMC7524089

[13] Levey AS, Stevens LA, Schmid CH, Zhang Y(L), Castro AF, Feldman HI et al. A new equation to estimate glomerular filtration rate. Ann Intern Med 2009;150:604–612. 10.7326/0003-4819-150-9-200905050-00006 19414839 PMC2763564

[14] Kidney disease: improving global outcomes (KDIGO) CKD work Group. KDIGO 2012 clinical practice guideline for the evaluation and management of chronic kidney disease. Kidney Inter Suppl 2013;3:1–150.

[15] Wettermark B, Hammar N, Michaelfored C, Leimanis A, Otterblad Olausson P, Bergman U et al. The new Swedish Prescribed Drug Register—opportunities for pharmacoepidemiological research and experience from the first six months. Pharmacoepidemiol Drug Saf 2007;16:726–735. 10.1002/pds.1294 16897791

[16] Baumgartner PC, Haynes RB, Hersberger KE, Arnet I. A systematic review of medication adherence thresholds dependent of clinical outcomes. Front Pharmacol 2018;9:1290. 10.3389/fphar.2018.01290 30524276 PMC6256123

[17] Prieto-Merino D, Mulick A, Armstrong C, Hoult H, Fawcett S, Eliasson L et al. Estimating proportion of days covered (PDC) using real-world online medicine suppliers' datasets. J Pharm Policy Pract 2021;14:113. 10.1186/s40545-021-00385-w 34965882 PMC8715592

[18] WHO Collaborating Centre for Drug Statistics Methodology , ATC Classification Index with DDDs. https://www.whocc.no/atc_ddd_index/. 2024

[19] Lindberg F, Benson L, Dahlström U, Lund LH, Savarese G. Trends in heart failure mortality in Sweden between 1997 and 2022. Eur J Heart Fail 2025;27:366–376. 10.1002/ejhf.3506 39463287 PMC11860728

[20] Kapelios CJ, Canepa M, Benson L, Hage C, Thorvaldsen T, Dahlström U et al. Non-cardiology vs. cardiology care of patients with heart failure and reduced ejection fraction is associated with lower use of guideline-based care and higher mortality: observations from The Swedish Heart Failure Registry. Int J Cardiol 2021;343:63–72. 10.1016/j.ijcard.2021.09.013 34517016

[21] Makubi A, Hage C, Sartipy U, Lwakatare J, Janabi M, Kisenge P et al. Heart failure in Tanzania and Sweden: comparative characterization and prognosis in the Tanzania Heart Failure (TaHeF) study and the Swedish Heart Failure Registry (SwedeHF). Int J Cardiol 2016;220:750–758. 10.1016/j.ijcard.2016.06.239 27393861 PMC5553107

[22] Kapelios CJ, Benson L, Crespo-Leiro MG, Anker SD, Coats AJS, Chioncel O et al. Participation in a clinical trial is associated with lower mortality but not lower risk of HF hospitalization in patients with heart failure: observations from the ESC EORP Heart Failure Long-Term Registry. Eur Heart J 2023;44:1526–1529. 10.1093/eurheartj/ehad109 36879413 PMC10149529

[23] Kapelios CJ, Lund LH. Preemptive versus urgent heart failure hospitalization as a surrogate for mortality risk in heart failure. Circulation 2024;149:1062–1064. 10.1161/circulationaha.123.068066 38557123

[24] Lindberg F, Lund LH, Benson L, Schrage B, Edner M, Dahlström U et al. Patient profile and outcomes associated with follow-up in specialty vs. primary care in heart failure. ESC Heart Fail 2022;9:822–833. 10.1002/ehf2.13848 35170237 PMC8934918

[25] Savarese G, Lund LH, Dahlström U, Strömberg A. Nurse-led heart failure clinics are associated with reduced mortality but not heart failure hospitalization. J Am Heart Assoc 2019;8:e011737. 10.1161/jaha.118.011737 31094284 PMC6585319

