# Supplementary material for: Characteristics, use of guideline–recommended medical therapies and clinical outcomes of patients with heart failure not enrolled in a quality registry: a comparison with the Swedish Heart Failure Registry
Source: Eur Heart J Qual Care Clin Outcomes. 2025 Mar 26;11(7):993–1003. doi: 10.1093/ehjqcco/qcaf019 (PMC12587275; doi:10.1093/ehjqcco/qcaf019)
Supplement: qcaf019_Supplemental_File [file qcaf019_supplemental_file.docx]

**Characteristics, use of guideline-recommended medical therapies and clinical outcomes of patients with heart failure not enrolled in a quality registry: A comparison with the Swedish Heart Failure Registry**

Ailema Gonzalez-Ortiz, Paul Hjemdahl, Faizan Mazhar, Alessandro Bosi , Anne-Laure Faucon, Gianluigi Savarese, Lars H Lund, Juan Jesus Carrero.

| METHODS……………………………………………………………………………………………………2  Table S1. Heart Failure definition, comorbidities and procedures …………………………………………….2  Table S2. Medications …………………………………....................................................................................3  Table S3. Clinical Outcomes …………………………......................................................................................3  Ejection fraction estimation ………………………………………………………………………………….4-5  Figure S1. Time since Heart Failure identification, until enrollment occurred………………….....................5  RESULTS …………………………………………………………………………………………………….6  Figure S2. Flowchart of study inclusion………………………………………...…………..............................6  Table S4 Sociodemographic and clinical characteristics or adults with incident heart failure in Stockholm,  Sweden, during 2012-2021, overall and by enrollment in the SwedeHF registry, along with univariable  Odds Ratio of enrollment (vs non-enrollment), setting of care…………........................................................7  Table S5. Unadjusted Odds Ratio of initiation of guideline-recommended within 180 days from diagnosis  and by enrollment in SwedeHF, those who survived 90 days and where enrolled in Swedehf  per setting……………………………………………………………………………………………………….8  Table S6. Multivariable adjusted odds ratios (OR) and 95% confidence intervals [CI] for persistence and  adherence of treatment *three years* years of guideline-recommended medical therapies within 90  days from identification. Shown are OR of SwedeHF-enrolled versus non-enrolled patients…………………8  SENSITIVITY ANALISIS ……………………………………………………………………………………..  Table S7. Unadjusted Odds Ratio of initiation of guideline-recommended within 30 , 90 and 180 days from diagnosis and by enrollment in SwedeHF of those who where enrolled in Swedehf within 180 days………….9  Table S8. Sensitivity Unadjusted Odds Ratio of initiation of guideline-recommended within 30 , 90 and 180  days from diagnosis and by enrollment in SwedeHF, those who survived 180 days and where enrolled in  Swedehf per setting………………………………………………………………………………………………9  Table S9. Multivariable-adjusted odds ratios (OR) and 95% confidence intervals [CI] for the adherence (Panel A) and persistence (Panel B) to therapies in SwedeHF-enrolled vs non-enrolled participants, overall and by setting of care…………………………………………………………………………………………………10  Table S10 . Adverse health outcomes associated with enrollment in SwedeHF (versus non-enrollment)…….11  Table S11 Sociodemographic and clinical characteristics of adults with incident heart failure with  reduced ejection fraction (HFrEF and HFmrEF; EF <50%) as determined by a Swedish-based  algorithm to estimate ejection fraction (EF)(2) , overall and by enrollment in the SwedeHF registry,  along with univariable Odds Ratio of enrollment (vs non-enrollment). ……………………………….…….13  Table S12. Unadjusted Odds Ratio for use of guideline-recommended therapy within 90 days from  diagnosis and by enrollment in SwedeHF. Participants with heart failure and mild-reduced ejection  fraction (<50%) …………………………………………………………………………………………………………………………………………14  TABLE S13. Multivariable-adjusted odds ratios (OR) and 95% confidence intervals [CI] for the adherence (Panel A) and persistence (Panel B) to therapies in SwedeHF-enrolled vs non-enrolled participants, overall HfmrEF participants ……………………………………………………………………………………………………………………………………..14 |
| --- |

**METHODS**

| Table S1. Heart Failure definition, comorbidities and procedures | |
| --- | --- |
| Heart Failure | I50, I50.1, I50.9, 142.0, I42.6, I42.7, I255, I110, I130, I132 between 2012-2021, and who survived 90 days from the first identification |
| Diabetes | ICD-E10-E14 |
| Hypertension | ICD-I10-I15 |
| Angina pectoris (within3 years) | ICD-120 |
| Myocardial infarction | ICD- I21, I22, I252 |
| Percutaneous coronary intervention | FNG (procedure code) |
| Coronary artery bypass graft | FNA, FNB, FNC, FND, FNE, FNF, FNH (Procedure codes)  ICD-10 codes Z951, Z955 |
| Stroke or TIA including hemorrhagic | I60-64, I690-694, G45 |
| Any severe bleed except intracranial | S064, S065, S066, I850, I983, K226, K250, K252, K254, K256, K260, K262, K264, K266, K270, K272 |
| Peripheral artery disease | I70-I73 |
| Aortic stenosis | I350, I352 |
| Aortic valve surgery | FMD10, FMD00, FMA32, FMD96, FMW96, FMA20, FMC96, FMD30, FMC00 (Procedure codes) |
| Atrial fibrillation/flutter | ICD-I48 |
| Devices (CRT, ICD, or pacemaker) | “Implantable cardioverter defibrillator or cardiac resynchronization therapy”:  ICD-10 Z950, Presence of electronic cardiac devices ICD-10 Z95810, Presence of other specified functional implants, Presence of automatic (implantable) cardiac defibrillator  ICD-10 Z450, Adjustment and management of cardiac devices  KVÅ FPE, Insertion or replacement of a permanent transvenous pacemaker  KVÅ FPF, Insertion or replacement of permanent epicardial pacemaker  KVÅ FPG, Insertion of permanent cardioverter-defibrillator  KVÅ DF016, Pacemaker |
| Lung disease | J4, J6-J9 |
| COPD | ICD-J40-J44 |
| Anemia | Haemoglobin < 130 g/L for men or < 120 g/L for women ICD CODES: D50-64, Z51.3, DR029, DR030, DR033, DR034 OR ATC B03A, B03X |

| Table S2. Medications | |
| --- | --- |
| ***HF medications*** |  |
| ACEi/ARB/ARNi: |  |
| RAS inhibitor  ACEi/ARB | ATC-C09A, C09B, C09C, C09D, C09X  ACE inhibitors, Angiotensin II receptor blockers (ARBs) and Renin inhibitors (including combinations) |
| RASi | C09(A-D) |
| ARNi | C09DX04 |
| Beta-blocker | C07 |
| Mineralocorticoids | C03DA  Spironolactone or Eplerenone |
| ***Other cardiovascular medications*** |  |
| Digoxin | C01AA05 |
| Calcium antagonists | C08CA, C08D |
| Statins | C10AA |
| Aspirin/anti-platelets | B01AC |
| Anticoagulants | B01AA, B01AE07, B01AF, B01AX05 |
| Diuretics | C03 |

**Table S3. OUTCOMES**

Definitions of MACE: mayor adverse cardiovascular events (composite cardiovascular death, nonfatal myocardial infarction, nonfatal ischemic stroke

| **Outcomes** | **Definition** |
| --- | --- |
| Cardiovascular death | ICD-10 code of the I family as main cause of death |
| Myocardial infarction | Hospitalization diagnosis with ICD-10 codes I20.0, I21, I22 in first or second diagnostic position |
| Ischemic stroke | Hospitalization diagnosis with ICD-10 code I63 in first or second diagnostic position |
| Hospitalization for heart failure | Hospitalization diagnosis with ICD-10 codes I50, I11.0, I13.0, I13.2, J81 in first or second diagnostic position |
|  |  |

**Ejection fraction estimation**

Left ventricular ejection fraction (EF) is required to categorize heart failure (HF) [i.e. HF with preserved (HFpEF), mid-range (HFmrEF), and reduced (HFrEF) EF] but is often not captured in population-based cohorts or non-HF registries. The aim of the algorithm is to identify EF subphenotypes for research purposes.

Reference

[1] Uijl A, Lund LH, Vaartjes I, Brugts JJ, Linssen GC, Asselbergs FW, et al. A registry-based algorithm to predict ejection fraction in patients with heart failure. ESC Hear Fail 2020;7:2388-2397. https://doi.org/10.1002/ehf2.12779.

Algorithm

Predictors in the model are: age (categorized as <75 and ≥75 years), sex (male/female), clinical characteristics, eGFR (<30, 30-60, 60-90 and ≥90 ml/min/1.73m^2^), comorbidities (yes vs. no), history of ischaemic heart disease, atrial fibrillation, chronic obstructive pulmonary disease [COPD], diabetes, hypertension, anemia, cancer in the previous 3 years, valvular disease, and use of treatments (yes vs. no), device therapy [implantable cardioverter defibrillator or cardiac resynchronization therapy], renin–angiotensin system [RAS] inhibitors, beta-blockers, diuretics, mineralocorticoid receptor antagonist [MRA], digoxin.

# Predicted risk calculation

For all patients with heart failure at baseline, EF was predicted with the following formula:

$$P\left( EF\geq50\% \right)=\frac{X}{1+X}$$

With

$X=exp(\ln\left( 0.32 \right)+\mathbb{1}_{age\geq75}*\ln\left( 1.58 \right)+\mathbb{1}_{sex=female}*\ln\left( 2.33 \right)+\mathbb{1}_{eGFR<30}*\ln\left( 0.60 \right)+\mathbb{1}_{eGFR 30-60}*\ln\left( 0.82 \right)+\mathbb{1}_{eGFR 60-90}*\ln\left( 0.87 \right)+\mathbb{1}_{ischaemic heart disease=yes}*\ln\left( 0.63 \right)+\mathbb{1}_{anemia=yes}*\ln\left( 1.44 \right)+\mathbb{1}_{atrial fibrillation=yes}*\ln\left( 1.45 \right)+\mathbb{1}_{COPD=yes}*\ln\left( 1.34 \right)+\mathbb{1}_{diabetes=yes}*\ln\left( 1.01 \right)+\mathbb{1}_{hypertension=yes}*\ln\left( 1.68 \right)+\mathbb{1}_{valvular disease=yes}*\ln\left( 1.31 \right)+\mathbb{1}_{malignant cancer=yes}*\ln\left( 1.18 \right)+\mathbb{1}_{device therapy=yes}*\ln\left( 0.30 \right)+\mathbb{1}_{RAS-inhibitor use=yes}*\ln\left( 0.40 \right)+\mathbb{1}_{beta-blocker use=yes}*\ln\left( 0.51 \right)+\mathbb{1}_{MRA use=yes}*\ln\left( 0.78 \right)+\mathbb{1}_{digoxin use=yes}*\ln\left( 0.91 \right)+\mathbb{1}_{diuretic use=yes}*\ln\left( 1.15 \right))$

Hereby, $\mathbb{1}$ is the indicator function, meaning that:

$$\mathbb{1}_{age\geq75}= \left\{ \begin{aligned} 1, if age\geq75 \\ 0, if age<75 \end{aligned} \right.$$

# Cut-off

A predicted probability threshold cut-off of 0.23 can be used to maximize the sensitivity and specificity of the model, as previously defined, creating the following EF phenotypes:

$$Predicted EF phenotype at baseline= \left\{ \begin{aligned} no HF at baseline ,if no HF at baseline \\ HFrEF or HFmrEF (predicted EF<50\%), if P\left( EF<50\% \right)<0.23 \\ HFpEF \left( predicted EF\geq50\% \right), , if P\left( EF\geq50\% \right)\geq0.23 \end{aligned} \right.$$

**Figure S1. Time between Heart Failure identification and SwedeHF enrollment.**


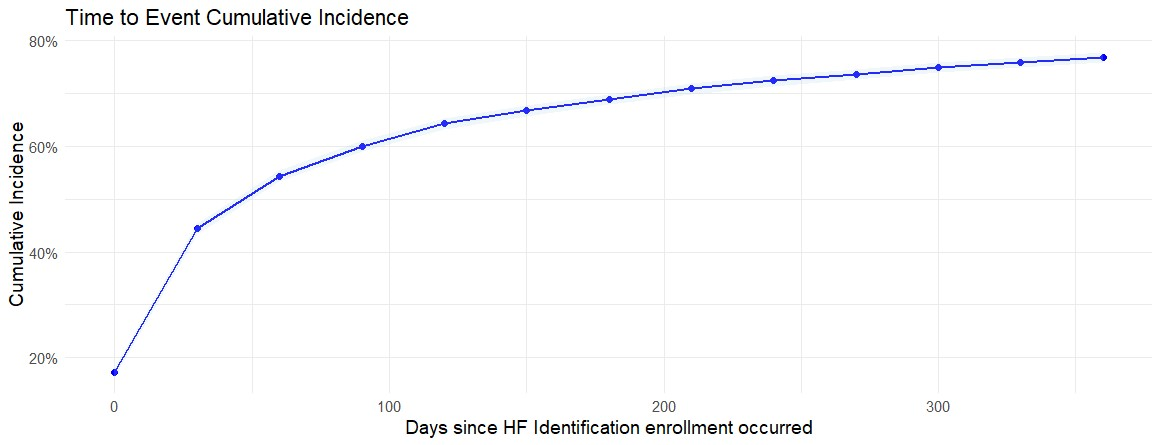


Figure S1. Shows that more than 60% of SwedeHF group were enrolled during the first 90 days

**RESULTS**

**Figure S2: Flowchart of study inclusion**

Selection of patient with heart failure diagnosis

Entire population with a HF diagnosis

N= 118850

Incident HF

(2012-2021)

n=55590

ENROLLED

n=4878

NON-ENROLLED

n= 43496

Excluded

n=195 < 18 years old

n=8 incorrect data

n= 7013 died

Study population

n= 48374

Requiring Hospitalization n=2903

Managed by Cardiology n=1831

Managed in Primary-Care n=144

Requiring Hospitalization n=20383

Managed by Cardiology n=9792

Managed in Primary-Care n=13321

Population with HF

n=118493

Excluded n=357

Incorrect diagnosis

**Table S4 Sociodemographic and clinical characteristics or adults with incident heart failure in Stockholm, Sweden,during 2012-2021, overall and by enrollment in the SwedeHF registry, along with univariable Odds Ratio of enrollment (vs non-enrollment), according by setting of care**

|  | *Non-enrolled*  *n=9792* | *Enrolled in SwedeHF*  *n=1831* | *Non-enrolled*  *n=20383* | *Enrolled in SwedeHF*  *n= 2903* | *Non-enrolled*  *n=13321* | *Enrolled in SwedeHF*  *n=144* |
| --- | --- | --- | --- | --- | --- | --- |
| **Setting** | Outpatient Cardiology | | Inpatient Cardiology | | Primary Care | |
| **Sociodemographic characteristics %** | | |  |  |  |  |
| Wome | 40 | 34 | 50 | 36 | 54 | 39 |
| Age years mean (SD) | 71.5 (13.2) | 68.4 (12.6) | 77.7 (12.9) | 69.8 (13.7) | 80.0 (10.4) | 77.0 (10.3) |
| >75 years ol | 47 | 36 | 66 | 41 | 75 | 65 |
| Living status |  |  |  |  |  |  |
| Living alone | 46 | 43 | 61 | 51 | 58 | 58 |
| Living with someone | 53 | 56 | 38.7 | 48.7 | 41 | 39 |
| Unknown | 1 | 1 | 0.3 | 0.3 | 1 | 2 |
| *Highest attained education* |  |  |  |  |  |  |
| Compulsory school | 26 | 22 | 33 | 28 | 32 | 27 |
| Secondary school | 41 | 42 | 40 | 43 | 39 | 38 |
| University | 32 | 33 | 24 | 27 | 25 | 29 |
| Unknown | 1 | 3 | 3 | 2 | 4 | 6 |
| *Estimated ejection fraction* |  |  |  |  |  |  |
| HFmrEF>=50% | 37 | 27 | 55 | 41 | 55 | 27 |
| HFEF < 50% | 56 | 65 | 29 | 33 | 37 | 56 |
| Unknown | 7 | 8 | 16 | 26 | 8 | 7 |
| **Comorbid conditions and history of procedures %** | |  |  |  |  |  |
| Hypertension | 68 | 62 | 73 | 55 | 79 | 58 |
| Chronic kidney disease | 30 | 25 | 40 | 29 | 40 | 52 |
| Diabetes | 23 | 22 | 26 | 22 | 24 | 17 |
| Myocardial infarction | 20 | 23 | 15 | 14 | 14 | 24 |
| Percutaneous coronary intervention | 18 | 19 | 11 | 11 | 10 | 16 |
| Coronary artery bypass graft | 5 | 6 | 4 | 4 | 4 | 8 |
| Angina pectoris (within 3 years) | 21 | 19 | 21 | 16 | 22 | 29 |
| Stroke or TIA including hemorrhagic | 17 | 13 | 21 | 13 | 21 | 13 |
| Any severe bleed except intracranial | 3 | 2 | 4 | 2 | 3 | 1 |
| Peripheral artery disease | 11 | 10 | 13 | 10 | 11 | 14 |
| Aortic stenosis | 6 | 5 | 7 | 5 | 5 | 8 |
| Aortic valve surgery | 3 | 2 | 2 | 2 | 2 | 2 |
| Atrial fibrillation | 43 | 41 | 39 | 31 | 44 | 51 |
| Lung disease | 31 | 29 | 33 | 28 | 32 | 26 |
| COPD | 15 | 13 | 18 | 12 | 18 | 17 |
| Recent Anemia (1 year) | 13 | 13 | 20 | 14 | 14 | 12 |
| ***HF specific medications* %** | | |  |  |  |  |
| *ACEi/ARB/ARNi* | 70 | 83 | 48 | 42 | 64 | 81 |
| *Beta-blocker* | 74 | 79 | 53 | 43 | 65 | 91 |
| *MRA* | 15 | 19 | 6 | 6 | 10 | 20 |
| ***Other cardiovascular medications* %** | |  |  |  |  |  |
| *Diuretics* | 47 | 50 | 70 | 29 | 60 | 67 |
| *Digoxin* | 7 | 7 | 4 | 3 | 6 | 10 |
| *Anticoagulants* | 40 | 42 | 28 | 24 | 37 | 64 |
| *Statins* | 46 | 47 | 32 | 29 | 38 | 49 |
| *Aspirin/antiplatelets* | 40 | 40 | 33 | 28 | 36 | 35 |
| *Calcium antagonists* | 27 | 24 | 29 | 22 | 33 | 21 |

Categorical variables are presented as percentages. Continuous variables are presented as mean (standard deviation) SD; standard deviation , TIA; transient ischemic attack, COPD; chronic obstructive pulmonary disease. 

**Table S5. Unadjusted Odds Ratio of initiation of guideline-recommended within 180 days from diagnosis and by enrollment in SwedeHF, those who survived 90 days and where enrolled in Swedehf per setting**

| n=48374 | Outpatient Cardiology  n=11623 | | | Inpatient Cardiology  n=23286 | | | Primary Care  n=13465 | | |
| --- | --- | --- | --- | --- | --- | --- | --- | --- | --- |
| *Medications* | *Non-enrolled, %* | *SwedeHF-enrolled,*  *%* | *Odds Ratio*  *(95% CI)* | *Non-enrolled, %* | *SwedeHF-enrolled, %* | *Odds Ratio*  *(95% CI)* | *Non-enrolled, %* | *SwedeHF-enrolled, %* | *Odds Ratio*  *(95% CI)* |
| ACEi/ARB/ARNi | 77 | 90 | 2.77 (2.36-3.26) | 72 | 91 | 3.68 (3.24 -4.19) | 67 | 84 | 2.54 (1.63-3.98) |
| *Beta-blocker* | 78 | 86 | 1.68(1.46-1.94) | 81 | 94 | 3.63 (3.11-4.23) | 67 | 92 | 5.35(2.96-9.66) |
| *MRA* | 24 | 36 | 1.85 (1.66-2.06) | 28 | 54 | 3.07 (2.84-3.32) | 14 | 24 | 2.05 (1.40-3.02) |

**Table S6. Multivariable adjusted odds ratios (OR) and 95% confidence intervals [CI] for persistence and adherence of treatment *three years* years of guideline-recommended medical therapies within 90 days from identification. Shown are OR of SwedeHF-enrolled versus non-enrolled patients.**

|  | Overall  n=48374 | | By Setting of care | | | |
| --- | --- | --- | --- | --- | --- | --- |
|  |  |  | Outpatient Cardiology  n=11623 | Inpatient Cardiology  n=23286 | Primary Care  n=13465 |  |
| ***Panel A. Three years adherence to guideline-recommended medical therapies*** | | | | | | |
|  | % of adherent, enrolled/non-enrolled | OR (95% CI) | OR (95% CI) | OR (95% CI) | OR (95% CI) | P for interaction |
| *ACEi/ARB/ARNi* | 93/87 | 1.64 (1.31 -2.06) | 1.74 (1.39-2.19) | 1.67 (1.40-2.00) | 2.66 (1.59-4.46) | 0.17 |
| *Beta-blocker* | 54/48 | 1.26 (1.12 -1.42) | 1.27 (1.13-1.43) | 1.20 (1.09-1.32) | 1.85 (1.42-2.40) | 0.11 |
| *MRA* | 37/38 | 1.03(0.92-1.15) | 1.50 (1.22-1.83) | 0.87 (0.76-1.00) | 1.45 (0.95-2.20) | 0.01 |
| ***Panel B. Three years persistence to guideline-recommended medical therapies*** | | | | | | |
|  | % of persistent, enrolled/non-enrolled | OR (95% CI) | OR (95% CI) | OR (95% CI) | OR (95% CI) | P for interaction |
| *ACEi/ARB/ARNi* | 87/76 | 1.59 (1.34-1.90) | 1.69 (1.42-2.01) | 1.81 (1.59-2.05) | 2.19 (1.51-3.19) | 0.88 |
| *Beta-blocker* | 69/60 | 1.43 (1.06-1.93) | 1.31 (1.15-1.48) | 1.54 (1.40-1.70) | 1.90 (1.43-2.53) | 0.69 |
| *MRA* | 46/37 | 1.99 (1.23-3.23) | 1.42 (1.17-1.74) | 1.31 (1.14-1.50) | 1.27 (0.82-1.96) | 0.14 |

**Sensitivity analysis**

**45225 Participans who survived at least 180 days**

**Table S7.** Unadjusted Odds Ratio of initiation of guideline-recommended within 30 , 90 and 180 days from diagnosis and by enrollment in SwedeHF of those who where enrolled in Swedehf within 180 days

| n=45225 | Within 30 days from diagnosis | | | Within 90 days from diagnosis | | | Within 180 days from diagnosis | | |
| --- | --- | --- | --- | --- | --- | --- | --- | --- | --- |
| *Medications* | *Non-enrolled, %* | *SwedeHF-enrolled,*  *%* | *Odds Ratio*  *(95% CI)* | *Non-enrolled, %* | *SwedeHF-enrolled, %* | *Odds Ratio*  *(95% CI)* | *Non-enrolled, %* | *SwedeHF-enrolled, %* | *Odds Ratio*  *(95% CI)* |
| ACEi/ARB/ARNi | 48 | 68 | 2.32 (2.19-2.47) | 67 | 86 | 3.21 (2.95-3.48) | 72 | 91 | (4.02 - 4.43) |
| *Beta-blocker* | 50 | 65 | 1.91 (1.80-2.03) | 70 | 86 | 2.73 (2.51-2.96) | 76 | 91 | 3.33 (3.02-3.68) |
| *MRA* | 13 | 28 | 2.56 (2.40-2.74) | 18 | 40 | 2.97(2.79 - 3.15) | 22 | 47 | 3.21(3.02-3.40) |

ACEi, angiotensin-converting enzyme inhibitor; ARB, angiotensin receptor blocker; ARNi, angiotensin receptor–neprilysin inhibitor; MRA, mineralocorticoid receptor antagonist. *ARNi initiations were considered for the period 2016-2021

**Table S8.** Sensitivity Unadjusted Odds Ratio of initiation of guideline-recommended within 30 , 90 and 180 days from diagnosis and by enrollment in SwedeHF, those who survived 180 days and where enrolled in Swedehf per setting

| N=45225 | CARDIOLOGY | | | INPATIENT | | | PRIMARY CARE | | |
| --- | --- | --- | --- | --- | --- | --- | --- | --- | --- |
| *Medications* | *Non-enrolled, %* | *SwedeHF-enrolled,*  *%* | *Odds Ratio*  *(95% CI)* | *Non-enrolled, %* | *SwedeHF-enrolled, %* | *Odds Ratio*  *(95% CI)* | *Non-enrolled, %* | *SwedeHF-enrolled, %* | *Odds Ratio*  *(95% CI)* |
| ACEi/ARB/ARNi | 77 | 91 | 3.04 (2.59-3.57) | 73 | 91 | 4.12 (3.61-4.71) | 68 | 85 | 2.63 (1.70-4.07) |
| *Beta-blocker* | 78 | 87 | 1.80 (1.57-2.07) | 81 | 94 | 3.93(3.36-4.60) | 67 | 92 | 5.79 (3.21-10.45) |
| *MRA* | 23 | 37 | 1.97 (1.77-2.18) | 27 | 55 | 3.22 (2.98-3.48) | 13 | 32 | 3.04(2.17 - 4.28) |

**Table S9.** Multivariable-adjusted odds ratios (OR) and 95% confidence intervals [CI] for the adherence (Panel A) and persistence (Panel B) to therapies in SwedeHF-enrolled vs non-enrolled participants, overall and by setting of care.

|  | Overall  n=45225 | | By Setting of care | | | |
| --- | --- | --- | --- | --- | --- | --- |
|  |  |  | Outpatient Cardiology  n=11066 | Inpatient Cardiology  n=21430 | Primary Care  n=12729 |  |
| ***Panel A. One year adherence to guideline-recommended medical therapies*** | | | | | | |
|  | % of adherent, enrolled/non-enrolled | OR (95% CI) | OR (95% CI) | OR (95% CI) | OR (95% CI) | P for interaction |
| *ACEi/ARB/ARNi* | 94/89 | 2.54(1.50-4.31) | 1.66(1.31-2.10) | 1.71(1.43-2.10) | 2.65(1.14-6.16) | 0.18 |
| *Beta-blocker* | 57/49 | 1.47(1.12-1.92) | 1.24(1.10-1.39) | 1.26(1.15-1.38) | 1.28(0.86-1.90) | 0.20 |
| *MRA* | 38/38 | 1.01(0.91-1.13) | 1.29(1.05-1.58) | 0.93 (0.82-1.07) | 1.43(0.68-3.05) | 0.03 |
| ***Panel B. One year persistence to guideline-recommended medical therapies*** | | | | | | |
|  | % of persistent, enrolled/non-enrolled | OR (95% CI) | OR (95% CI) | OR (95% CI) | OR (95% CI) | P for interaction |
| *ACEi/ARB/ARNi* | 93/86 | 1.37(0.85-2.22) | 1.58(1.26-1.99) | 2.19(1.87-2.56) | 1.11(0.60-2.08) | 0.08 |
| *Beta-blocker* | 85/76 | 1.46(1.01-2.11) | 1.46(1.25-1.72) | 1.71(1.53-1.92) | 2.03(1.15-3.61) | 0.43 |
| *MRA* | 65/53 | 1.49(0.91-2.44) | 1.37(1.11-1.70) | 1.39(1.22-1.59) | 1.43(0.67-3.06) | 0.89 |

ACEi, angiotensin-converting enzyme inhibitor; ARB, angiotensin receptor blocker; ARNi, angiotensin receptor–neprilysin inhibitor; MRA, mineralocorticoid receptor antagonist. *We create a model per each medication, adjusted for clinical characteristics, and comorbidity

**Table S10 .** Adverse health outcomes associated with enrollment in SwedeHF (versus non-enrollment).

|  | Overall  n=45225 | | | By Setting of care | | | |
| --- | --- | --- | --- | --- | --- | --- | --- |
|  |  |  |  | Outpatient Cardiology  n=11066 | Inpatient Cardiology  n=21430 | Primary Care  n=12729 |  |
|  | *No. events/cases*  Non-enrolled | *No. events/cases*  Enrolled | HR (95% CI) | HR (95% CI) | HR (95% CI) | HR (95% CI) | P for interaction |
| ***MACE*** |  |  |  |  |  |  |  |
| SwedeHF-enrolled | 10900/39976 | 1171/5249 | 0.94(0.88-1.01) | 0.90(0.80-1.03) | 0.95(0.88-1.02) | 1.11(0.83-1.49) | <0.01 |
| **HF hospitalization** |  |  |  |  |  |  |  |
| SwedeHF-enrolled | 14038/39976 | 2195/5429 | 1.32(1.26-1.39) | 1.20)1.09-1.32) | 1.33(1.26-1.41) | 1.91(1.50-2.43) | <0.01 |
| **All-cause mortality** |  |  |  |  |  |  |  |
| SwedeHF-enrolled | 15774/39976 | 1438/5429 | 0.89(0.84-0.94) | 0.99(0.89-1.13) | 0.85(0.81-0.94) | 0.90(0.70-1.18) | <0.01 |

Output from Cox-Regression models adjusted for sociodemographic variables, history of comorbidities, guideline-recommended therapies (ACEi/ARB/ARNi, Beta-blocker, MRA) and, when applicable, setting of care.

**Subgroup analysis**

**Adults with incident heart failure with mild-reduced ejection fraction (HFmrEF, EF <50%) as estimated by a SwedeHF-based algorithm based on claims data (1)**

| **Table S11** **Sociodemographic** and clinical characteristics of adults with incident heart failure with reduced ejection fraction (HFrEF and HFmrEF; EF <50%) as determined by a Swedish-based algorithm to estimate ejection fraction (EF)(2) , overall and by enrollment in the SwedeHF registry, along with univariable Odds Ratio of enrollment (vs non-enrollment). | | | | |
| --- | --- | --- | --- | --- |
| **Sociodemographic characteristics %** | **HFrEF/HFmrEF cases** | **Non-enrolled** | **Enrolled in SwedeHF** | **Odds Ratio of enrollment** |
| Characteristics | n=18639 | n=16412 | n=2227 |  |
| Women | 21 | 21 | 19 | 0.85 (0.76-0.95) |
| Age years mean (SD) | 73 (12) | 74 (12) | 69 (12) | 0.97 (0.97-0.97) |
| >75 years old | 48 | 50 | 34 | 0.51 (0.46-0.56) |
| *Living status* |  |  |  |  |
| Living alone | 45 | 45 | 42 | 0.87 (0.79-0.95) |
| Living with someone | 55 | 54 | 58 | 1.14 (1.05-1.25) |
| Unknown | 0.3 | 0.3 | 0.5 | --- |
| *Highest attained education* |  |  |  |  |
| Compulsory school | 27 | 27 | 25 | 0.86 (0.78-0.95) |
| Secondary school | 42 | 41 | 42 | 1.05 (0.96-1.14) |
| University | 29 | 29 | 31 | 1.11 (1.00-1.22) |
| **Setting of identification and care** % | |  |  |  |
| Cardiology inpatient care | 37 | 36 | 43 | 1.31 (1.20-1.44) |
| Cardiology outpatient care | 36 | 34 | 53 | 2.26 (2.07-2.47) |
| Primary Care | 27 | 30 | 4 | 0.09 (0.07-0.11) |
| **Comorbid conditions and history of procedures %** | |  |  |  |
| Hypertension | 74 | 76 | 64 | 0.57 (0.52-0.62) |
| Chronic kidney disease | 33 | 34 | 26 | 0.70 (0.63-0.77) |
| Diabetes Mellitus | 31 | 32 | 28 | 0.82 (0.74-0.91) |
| Myocardial infarction | 27 | 27 | 27 | 1.00 (0.91-1.11) |
| Percutaneous coronary intervention | 22 | 22 | 23 | 1.05 (0.94-1.16) |
| Coronary artery bypass graft | 8 | 8 | 7 | 0.96 (0.81-1.14) |
| Angina pectoris (within 3 years) | 32 | 33 | 26 | 0.73 (0.66-0.81) |
| Stroke or TIA including hemorrhagic | 19 | 20 | 15 | 0.73 (0.65-0.82) |
| Any severe bleed except intracranial | 3 | 3 | 2 | 0.63 (0.47-0.85) |
| Peripheral artery disease | 13 | 13 | 12 | 0.86 (0.75-0.99) |
| Aortic stenosis | 5.5 | 6 | 5 | 0.81 (0.66-1.00) |
| Aortic valve surgery | 2.5 | 2.5 | 2.4 | 0.97 (0.73-1.29) |
| Atrial fibrillation | 40 | 41 | 38 | 0.89 (0.82-0.98) |
| Lung disease | 28 | 28 | 28 | 0.99 (0.90-1.09) |
| COPD | 12.5 | 13 | 12 | 0.94 (0.82-1.08) |
| Recent anemia (1 year) | 11.3 | 11 | 11 | 0.99 (0.86-1.14) |
| ***HF specific medications* %** |  |  |  |  |
| *ACEi/ARB/ARNi* | 85 | 85 | 86 | 1.01 (0.89-1.14) |
| *Beta-blocker* | 82 | 82 | 81 | 0.92 (0.82-1.03) |
| *MRA* | 14 | 13 | 17 | 1.38 (1.22-1.55) |
| ***Other cardiovascular medications* %** | |  |  |  |
| *Diuretics* | 48 | 48 | 46 | 0.91 (0.84-1.00) |
| *Digoxin* | 6 | 6 | 5 | 0.84 (0.69-1.03) |
| *Anticoagulants* | 37 | 37 | 37 | 1.00 (0.91-1.09) |
| *Statins* | 54 | 54 | 52 | 0.93 (0.85-1.02) |
| *Aspirin/antiplatelets* | 47 | 48 | 46 | 0.93 (0.85-1.01) |
| *Calcium channel blockers* | 33 | 33 | 28 | 0.78 (0.70-0.86) |
| Categorical variables are presented as percentages. Continuous variables are presented as mean (standard deviation) SD; standard deviation, TIA; transient ischemic attack, COPD; chronic obstructive pulmonary disease. | | | | |

**Table S12.** Unadjusted Odds Ratio for use of guideline-recommended therapy within 90 days from diagnosis and by enrollment in SwedeHF. Participants with heart failure and mild-reduced ejection fraction (<50%)

| HFmrEF cases n=18639 | Within 90 days from diagnosis | | |
| --- | --- | --- | --- |
| ***Medications*** | *Non-enrolled, %* | *SwedeHF-enrolled, %* | *Odds Ratio*  *(95% CI)* |
| **ACEi/ARB/ARNi** | 79 | 88 | 1.92 (1.68-2.20) |
| ***Beta-blockers*** | 78 | 87 | 1.82 (1.60-2.06) |
| ***MRA*** | 21 | 39 | 2.45 (2.23-2.68) |

ACEi, angiotensin-converting enzyme inhibitor; ARB, angiotensin receptor blocker; ARNi, angiotensin receptor–neprilysin inhibitor; MRA, mineralocorticoid receptor antagonist. *ARNi initiations were considered for the period 2016-2021

**TABLE S13****.** Multivariable-adjusted odds ratios (OR) and 95% confidence intervals [CI] for the adherence (Panel A) and persistence (Panel B) to therapies in SwedeHF-enrolled vs non-enrolled participants, overall HfmrEF participants

|  | HFmrEF cases n=18639 | |
| --- | --- | --- |
|  |  |  |
| ***Panel A. One year adherence to guideline-recommended medical therapies*** | | |
|  | **% adherent enrolled/non-enrolled** | **OR (95% CI)** |
| *ACEi/ARB/ARNi* | 94/91 | 2.31(1.12-4.78) |
| *Beta-blocker* | 56/49 | 1.53(1.07-2.21) |
| *MRA* | 41/37 | 1.23(1.05-1.45) |
| ***Panel B. One year persistence to guideline-recommended medical therapies*** | | |
|  | **% persistent enrolled/non-enrolled** | **OR (95% CI)** |
| *ACEi/ARB/ARNi* | 95/91 | 1.13 (0.56-2.27) |
| *Beta-blocker* | 88/82 | 1.29 (0.77-2.14) |
| *MRA* | 69/61 | 1.40 (0.72-2.73) |

ACEi, angiotensin-converting enzyme inhibitor; ARB, angiotensin receptor blocker; ARNi, angiotensin receptor–neprilysin inhibitor; MRA, mineralocorticoid receptor antagonist. *We create a model per each medication, adjusted for clinical characteristics (age, gender and when applicable setting of care), and comorbidities (Hypertension, Chronic kidney disease, Diabetes Mellitus, Myocardial infarction, Percutaneous coronary intervention, Coronary artery bypass graft, Angina pectoris, Stroke , Severe bleed , Aortic stenosis , Aortic valve surgery, Atrial fibrillation, Lung disease, COPD ,anemia)
